# Molecular characterization of glucose-6-phosphate dehydrogenase deficient variants in Baghdad city - Iraq

**DOI:** 10.1186/1471-2326-12-4

**Published:** 2012-03-27

**Authors:** Bassam MS Al-Musawi, Nasir Al-Allawi, Ban A Abdul-Majeed, Adil A Eissa, Jaladet MS Jubrael, Hanan Hamamy

**Affiliations:** 1 Department of Pathology, College of Medicine, University of Baghdad, Baghdad, Iraq; 2 Department of Pathology, College of Medicine, University of Dohuk, Azadi Hospital road, 1014 AM Dohuk, Iraq; 3 Scientific Research Center, University of Dohuk, Dohuk, Iraq; 4 Department of Genetic Medicine and Development, Geneva University Hospital, Geneva, Switzerland

**Keywords:** G6PD deficiency, Arabs, Baghdad, Iraq, G6PD Mediterranean, G6PD Chatham

## Abstract

**Background:**

Although G6PD deficiency is the most common genetically determined blood disorder among Iraqis, its molecular basis has only recently been studied among the Kurds in North Iraq, while studies focusing on Arabs in other parts of Iraq are still absent.

**Methods:**

A total of 1810 apparently healthy adult male blood donors were randomly recruited from the national blood transfusion center in Baghdad. They were classified into G6PD deficient and non-deficient individuals based on the results of methemoglobin reduction test (MHRT), with confirmation of deficiency by subsequent enzyme assays. DNA from deficient individuals was studied using a polymerase chain reaction-Restriction fragment length polymorphism (PCR-RFLP) for four deficient molecular variants, namely G6PD Mediterranean (563 C→T), Chatham (1003 G→A), A- (202 G→A) and Aures (143 T→C). A subset of those with the Mediterranean variant, were further investigated for the 1311 (C→T) silent mutation.

**Results:**

G6PD deficiency was detected in 109 of the 1810 screened male individuals (6.0%). Among 101 G6PD deficient males molecularly studied, the Mediterranean mutation was detected in 75 cases (74.3%), G6PD Chatham in 5 cases (5.0%), G6PD A- in two cases (2.0%), and G6PD Aures in none. The 1311 silent mutation was detected in 48 out of the 51 G6PD deficient males with the Mediterranean variant studied (94.1%).

**Conclusions:**

Three polymorphic variants namely: the Mediterranean, Chatham and A-, constituted more than 80% of G6PD deficient variants among males in Baghdad. Iraq. This observation is to some extent comparable to other Asian Arab countries, neighboring Turkey and Iran.

## Background

Glucose-6-Phosphate Dehydrogenase (G6PD) deficiency is the most common human enzyme deficiency affecting more than 400 million people worldwide [1].

The G6PD gene is located on the Xq28 region of the X chromosome and is about 20 kb in length comprising 13 exons and 12 introns [2]. Although G6PD is a house keeping enzyme that is expressed in all tissues, clinical manifestations of its deficiency are seen almost exclusively in red cells (RBC) including: neonatal jaundice and acute hemolytic anemia related to drugs, infection, or the ingestion of fava beans [2,3]

G6PD enzyme (G6PD) is known to protect RBCs from the harmful effects of reactive oxygen species. Mutations in G6PD gene that reduce the amount of G6PD enzyme or alter its structure cause hemolytic anemia as a result of accumulation of reactive oxygen species [2]. More than 200 mutations have been reported showing different distributions in different populations, with sharing of specific mutations within each population [4].

G6PD deficiency has been reported in almost all racial groups with prevalence rates ranging from less than 1% in Japan and Northern European populations to a high of 58% in Kurdish Jews [5-7]. High rates of G6PD deficiency have been reported from the Mediterranean Littoral, Middle East, Africa and south and south East Asia [1].

Previous studies on G6PD deficiency in Iraq focused on the prevalence rates in various parts of the country and among its two major ethnic groups namely: the Arabs and Kurds [8-12]. Only one study done on the Kurds of northern Iraq tackled the molecular aspects of G6PD-deficient variants [12]. The aim of the current study is to determine the prevalence of G6PD deficiency and characterize the deficient variants and their enzyme levels among asymptomatic healthy blood donors in the Arab population of central Iraq and to compare the findings with those reported among the Kurds in the North as well as among neighboring countries.

## Methods

A total of 1810 healthy adult male blood donors were randomly recruited from the National Blood Transfusion Center in Baghdad, Iraq from 28^th ^April to 26^th ^August 2008. They were classified into G6PD deficient and G6PD non-deficient individuals according to the result of Methemoglobin Reduction Test (MHRT) [13]. Deficiency was subsequently confirmed by quantitative enzyme assays according to the manufacturer instructions (Biolabo-France). G6PD enzyme assays were also performed on an additional 49 random G6PD non-deficient individuals as controls.

DNA from deficient cases was extracted from whole blood by phenol-chloroform method. The extracted DNA was screened sequentially for four G6PD deficient mutations namely G6PD Mediterranean (563 C→T), G6PD Chatham (1003 G→A), G6PD A- (202 G→A) and G6PD Aures (143 T→C). A subset of those with the Mediterranean variant were then screened for the silent (1311 C→T) mutation using a polymerase chain reaction/restriction fragment length polymorphism (PCR/RFLP) based method [12].

This research was approved by the ethical committee at the college of Medicine, University of Baghdad, Baghdad-Iraq, and informed consents were obtained from all participants.

Statistical analysis utilized the Mann Whitney U test, and a p < 0.05 was considered significant.

## Results

Among 1810 individuals enrolled in this study, 109 (6.0%) were found to be deficient by MHRT and then confirmed by enzyme assays.

Adequately extracted DNA samples from 101 of 109 deficient cases were included in further molecular studies. The remaining 8 samples were either inadequate or had failed DNA extraction.

Among 101 G6PD deficient males, the Mediterranean variant (563 C→T) was detected in 75 (74.3%), G6PD Chatham (1003 G→A) in 5 (5.0%), and G6PD A- (202 G→A) in 2 (2.0%) individuals. None of the remaining 19 cases showed the G6PD Aures (143 T→C) when tested for this variant. Table 1 outlines G6PD enzyme levels among those with the Mediterranean, Chatham and A- variants as well as the non-deficient controls. The enzyme levels were least among the Mediterranean, and they were significantly lower than those with the Chatham variant (*p *= 0.001).

**Table 1:** The G6PD enzyme levels (in IU/g Hb) in the three deficient variants identified in the current study and the control non-deficient group

Variant	Number	Range	Mean ± SD	Median
Mediterranean	75	0.16-0.89	0.35+0.14	0.35

Chatham	5	0.44-1.17	0.73+0.28	0.71

A-	2	2.10, 1.75		

Non-deficient controls	49	8.11-15.1	11.27+2.16	11.20

Among the 101 deficient individuals screened in this study, only one person with the Mediterranean variant gave a history suggestive of an acute hemolytic episode in his childhood, following the ingestion of fava beans (favism). Family history, however, indicated that in 15 individuals with the Mediterranean variant and 2 with the Chatham variant, there were episodes suggestive of acute hemolysis in one or more of their family members.

Among 51 cases with the Mediterranean variant, 48 (94.1%) showed the presence of the silent 1311 mutation (1311 C→T).

## Discussion

Baghdad lying at the center of Iraq was established as the Capital at the time of the Abbasid Empire (762-1258 AD). Current day Iraq coincides with ancient Mesopotamia that is labeled "the Cradle of Civilization" having witnessed some of the most ancient civilizations dating back to 7000 years. This fertile and rich land was throughout its history an attraction for invaders including Persians, Greeks, Romans, Mongols and Turks with variable impact on the mainly Arab population of Iraq [14].

**Figure 1:**
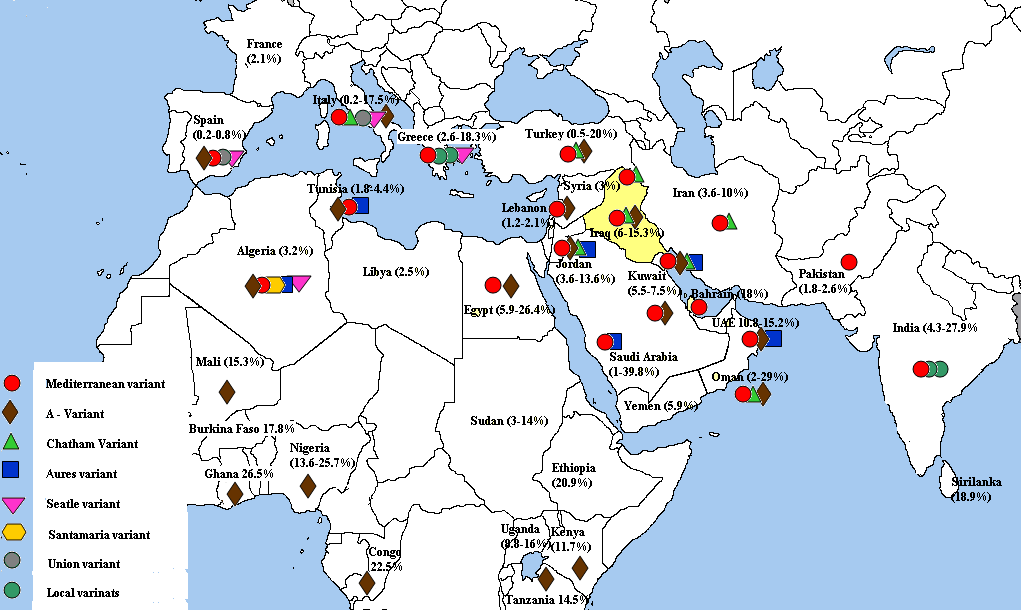
**The prevalence rates of G6PD deficiency and the most common deficient variants among males in Iraq and some of the surrounding populations **[6,8-12,16-32,38,42-56].

G6PD deficiency has long been recognized as a common inherited hematological disorder among Iraqis [8,9] with reported rates of 6.0-15.3% [8-12,15]. This study gave a rate of 6.0% among males in Baghdad, a figure that lies closer to the lower limit of previously reported range in this city of 6.1-12.3% [8-10]. This might be explained by the nature of this study where the subjects were male blood donors; it is expected that blood donation is not a common practice among individuals who could have experienced the hemolytic anemia of G6PD deficiency. Studies from Arab and neighboring countries showed a remarkable variation in the frequency of G6PD deficiency, even within the same country (Figure 1) [6,16]. Such variation maybe attributed to variable G6PD gene flow from Southern Europe and Africa, as well as on the presence of ethnic variations and the endemicity of malaria. G6PD deficiency is known to offer selective heterozygote advantage against certain malaria species in endemic areas [1,2].

The current study revealed that the most common G6PD deficient molecular variant was G6PD Mediterranean detected in 74.3% of deficient males. The Mediterranean mutation is the most common mutation in Asian Arab countries and Egypt, with frequencies ranging from a low of 53.6% in Jordan to a high of 91.2% in Bahrain [17-22], while it is the second most common variant in some African Arab countries like Algeria and Tunisia with frequencies of 23% and 11.4% respectively [23,24]. The Mediterranean mutation is also the most frequent mutation among Iraqi Kurds (87.8%), in neighboring Turkey (80%), Iran (66.2%-91.2%), Southern Europe (69-77%) and the Indian subcontinent (60.4-79.6%) [12,25-32] (Figure 1). The mutation decreases in frequency as we move east, though it is still present in polymorphic frequencies in Malaysia and Indonesia [33,34]. The association of the Mediterranean mutation with a silent 1311 mutation (1311 C→T) noted in Arab and other countries in the Middle East as well as southern Europe [21-23,35,36], is consistent with the notion of the common origin of this mutation in the Mediterranean basin within the past 1600 to 6640 years, and thereafter spreading to Middle east, and north Africa in the first millennium BC, where it was selected for by malaria already highly endemic in these fertile agricultural lands, particularly around 500 BC and thereafter [37]. In contrast to the latter, studies from Indian subcontinent (India and Pakistan) have documented that their Mediterranean mutation is much more likely to be associated with 1311C, suggesting a separate origin of the mutation in that part of the world [29,30,36].

The second most frequent variant detected in this study was G6PD Chatham, which was found in 5.0% of G6PD deficient individuals. Studies from other Arab countries showed variable rates of 10.1% in Kuwait, 3.6% in Jordan and 1% in Algeria [17,18,23], while it was encountered in 8.7% of Iraqi Kurds, 4% in Turkey and 2.2-27% in Iran[12,25-28]. G6PD Chatham is now recognized as one of the common variants worldwide [17,23,26], and in addition to Mediterranean countries, it has also been reported in polymorphic frequencies in Spain, India, Malaysia and Indonesia [29,33,38,39].

In this study, the African A- variant was present in only 2% of deficient males, a rate that is lower than the figures reported in some Asian Arab countries, where it is the second most common variant with rates of 5.8-16.7% [17-20]. The A- variant is the most frequent variant in some African Arab countries like Tunisia and Algeria with rates of 63.6% and 46% respectively [23,24]. The latter is anticipated since the A- mutation is almost the sole mutation responsible for G6PD deficiency in tropical Africa (Figure 1) and it is common in areas where people of African origin are present [2]. Our earlier study on Iraqi Kurds failed to document any case with the A- variant, while reports from neighboring Turkey and Iran reported rates of 2% and 0-1.35% respectively [12,25-28]. Such variability in the distribution of the African A- mutation could be explained by the different gene flow of this variant in African and Asian Arab countries. Future studies in Southern Iraq, where African gene flow is historically documented, may show results similar to those found in the Arabian Peninsula.

G6PD Aures was first described in an Algerian boy in 1993 [40], and later found to account for 7% of deficient variants in Algeria [23]. Thereafter, reports on this variant appeared from several Arab countries including Tunisia, Jordan, Kuwait, UAE and Western Saudi Arabia with frequencies of 4.5, 3.6, 3.0, 16.7 and 17% respectively [17,18,20,24,41]. None of the 19 uncharacterized cases in the current study showed this variant.

An intriguing observation relevant to the clinical phenotype associated with the Mediterranean variant (known to be a class II severe variant) in the current study is that only one individual (1.3%) with the latter variant had a personal history suggestive of favism. This should be viewed in the context of the design of the current study, which is based on screening apparently healthy individuals (blood donors), and maybe due to several causes including the possibility that some donors have had mild unnoticeable hemolytic episodes, or that those with documented favism may have refrained from blood donation voluntarily. Moreover, it is well known that hemolysis following bean consumption is not a rule, with a lot of individual variations even in the same individual at various times, in addition to the possibility of yet unidentified genetic factors that may play a role [1-3]. Our observation is shared by other studies of comparable design from other populations with predominance of the Mediterranean variant, including Saudi Arabia and Greece [32,42]. In his study including 757 apparently healthy male volunteers from Eastern Saudi Arabia, Al-Ali did not find any history of favism in any of his G6PD deficient subjects, despite the fact that they constituted 36.5-45.9% of the screened individuals, and that the Mediterranean variant is known to be their main deficient variant [19,42]. Another study from Greece which included screening 7,680 healthy adult males, found that 299 were G6PD deficient with the Mediterranean constituting 77.3% of the variants, yet none was symptomatic despite quite low enzyme levels [32]. The results of our study and those reviewed above favor the notion that many G6PD deficient individuals in these populations may remain asymptomatic throughout their lives, unaware of their status [2].

## Conclusions

Three polymorphic variants, namely the Mediterranean, Chatham and A- constituted the bulk of the G6PD deficient variants among the sample population in Baghdad, a result that is comparable to findings in neighboring Arab and non-Arab countries. The results of this study on Iraqi Arabs complement those of our earlier study on Iraqi Kurds, to give a more comprehensive idea about the distribution of G6PD variants in Iraq [12]. An important observation of the current study is that a significant number (~19%) of G6PD deficient cases remained uncharacterized, compared to around ~3% in our earlier study on Kurds [12], which is quite interesting and may reflect the much open admixture with other civilizations throughout the centuries [14]. DNA sequencing is necessary to determine whether these uncharacterized mutations were carried by gene flow or they represent novel mutations.

## Competing interests

The authors declare that they have no competing interests.

## Authors' contributions

BMSA, contributed to collection of data, performing larger part of the molecular studies, hematological and enzyme assays, data analysis and drafting of the manuscript; NA, contributed the concept and design, part of the molecular studies, data analysis and drafting of the manuscript; BAA contributed to the analysis and interpretation of data, drafting of the manuscript; AAE, contributed to part of the molecular work and analysis of data; JMSJ, contributed to the concept and design and part of the molecular work; HH, contributed to the analysis and interpretation of results, drafting and revision of the manuscript. All authors revised and approved the final submitted version of the manuscript.

## Pre-publication history

The pre-publication history for this paper can be accessed here:

http://www.biomedcentral.com/1471-2326/12/4/prepub

## References

[B1] Beutler E G6PD deficiency Blood 1994 84 3613 3636 7949118

[B2] Cappellini MC Fiorelli G Glucose-6-phosphate dehydrogenase deficiency Lancet 2008 371 64 74 10.1016/S0140-6736(08)60073-2 18177777

[B3] Mehta A Mason PJ Vulliamy TJ Glucose-6-phosphate dehydrogenase deficiency Bailliere Clinical Hematology 2000 13 21 38 10.1053/beha.1999.0055 10916676

[B4] Beutler E Vulliamy TJ Hematologically important mutations: glucose-6-phosphate dehydrogenase Blood Cells Mol Dis 2002 28 93 103 10.1006/bcmd.2002.0490 12064901

[B5] Nakatsuji T Miwa S Incidence and characteristics of glucose-6-phosphate dehydrogenase variants in Japan Hum Genet 1979 51 297 305 511159 10.1007/BF00283398

[B6] Nikhoma ET Poole C Vannappagari V Hall SA Beutler E The Global prevalence of glucose-6-phosphate dehydrogenase deficiency: A systemic review and meta-analysis Blood Cells Mol Dis 2009 42 267 278 10.1016/j.bcmd.2008.12.005 19233695

[B7] Oppenheim A Lury CL Rund D Vulliamy TJ Luzzatto L G6PD Mediterranean accounts for the high prevalence of G6PD deficiency in Kurdish Jews Hum Genet 1993 91 293 294 8478015 10.1007/BF00218277

[B8] Amin-Zaki L Taj Al-Din S Kubba K Glucose-6-phosphate dehydrogenase deficiency among ethnic groups in Iraq Bull World Health Organ 1972 47 1 1 5 4538901 PMC2480812

[B9] Hamamy H Saeed T Glucose-6-phosphate dehydrogenase deficiency in Iraq Hum Genet 1981 58 434 435 7327567 10.1007/BF00282832

[B10] Hilmi FA Al-Allawi NA Rassam M Al-Shamma G Al-Hashimi A Red cell glucose-6-phosphate dehydrogenase phenotypes in Iraq East Mediterr Health J 2002 8 1 6 15330559

[B11] Hassan MK Taha JY Al-Naama LM Widad NM Jasim SN Frequency of haemoglobinopathies and glucose-6-phosphate dehydrogenase deficiency in Basra East Mediterr Health J 2003 9 1 7 15562732

[B12] Al-Allawi N Eissa AA Jubrael JMS Jamal SAR Hamamy H Prevalence and Molecular Characterization of Glucose-6-Phosphate Dehydrogenase Deficient Variants among the Kurdish population of Northern Iraq BMC Blood Disorders 2010 10 6 10.1186/1471-2326-10-6 20602793 PMC2913952

[B13] Brewer GJ Tarlove AR Alving AS The methemoglobin reduction test from primaquine-type sensitivity to erythrocytes JAMA 1962 180 386 388 10.1001/jama.1962.03050180032008 13872968

[B14] Encyclopedia Britannica http://www.britannica.com/EBchecked/topic/293631/Iraq/214199/History

[B15] Eissa AA Muhammad FA Mohammed AI Al-Allawi NA Jalal SD Jubrael JMS Prevalence and molecular characterization of G6PD deficient variants in Sulymania province-Iraq Duhok Med J 2011 5 69 75

[B16] White JM Byrne M Richards R Buchanan T Katsoulis E Weerasingh K Red cell genetic abnormalities in Peninsular Arabs: sickle haemoglobin, G6PD deficiency, and alpha and beta thalassaemia J Med Genet 1986 23 245 251 10.1136/jmg.23.3.245 3723553 PMC1049636

[B17] Karadsheh NS Moses L Ismail SI Devaney JM Hoffman E Molecular heterogeneity of glucose-6-phosphate dehydrogenase deficiency in Jordan Haematologica 2005 90 1693 1694 16330444

[B18] AlFadhli S Kaaba S Elshafey A Salim M AlAwadi A Bastaki L Molecular characterization of glucose-6-phosphate dehydrogenase gene defect in the Kuwaiti population Arch Path Lab Med 2005 129 1144 1147 16119988 10.5858/2005-129-1144-MCOGDG

[B19] Al-Ali AK Al-Mustafa ZH Al-Madan M Qaw F Al-Ateeq S Molecular characterization of glucose-6-phosphate dehydrogenase deficiency in the eastern Province of Saudi Arabia Clin Chem Lab Med 2002 40 814 816 12392311 10.1515/CCLM.2002.141

[B20] Bayoumi RA Nur-E-Kamal MS Tadayyon M Mohamed KK Mahboob BH Qureshi MM Lakhani MS Awaad MO Kaeda J Vulliamy TJ Luzzatto L Molecular characterization of erythrocyte glucose-6-phosphate dehydrogenase deficiency in Al-Ain District, United Arab Emirates Hum Hered 1996 46 136 141 10.1159/000154342 8860007

[B21] Arnaout NH El-Gharbawy NM Shaheen IA Afifi RA Abd El-Dayem OY Incidence and Association of 563 C/T Mediterranean and the Silent 1311 C/T mutations in G6PD-deficient Egyptian Children LabMedicine 2011 42 355 360

[B22] Al-Momen N Al Arrayed SS Al Allawi A Molecular homogeneity of GPD Deficiency Bahrain Medical Bulletin 2004 26 1 6

[B23] Nafa K Reghis A Osmani N Baghli L Ait-Abbes H Benabadji M Kaplan J-C Vulliamy T Luzzatto L At least five polymorphic variants account for the prevalence of glucose 6-phosphate deficiency in Algeria Hum Genet 1994 94 513 517 7959686 10.1007/BF00211017

[B24] Ben Daoud B Mosbehi I Prehu C Chaouachi D Hafsia R Abbes S Molecular characterization of erythrocyte glucose-6-phosphate dehydrogenase deficiency in Tunisia Pathol Biol 2008 56 260 267 10.1016/j.patbio.2007.08.009 18226470

[B25] Oner R Gumruk F Acar C Oner C Gurgey A Altay C Molecular characterization of glucose-6- phosphate dehydrogenase deficiency in Turkey Haematologica 2000 85 320 321 10702825

[B26] Mesbah-Namin SA Sanati MH Momjoodi AR Mason PJ Vullamy TJ Noori-Daloii MR Three major glucose-6-phosphate dehydrogenase deficient polymorphic variants identified in Mazandaran state of Iran Brit J Haematol 2002 117 763 764 10.1046/j.1365-2141.2002.03483.x 12028056

[B27] Rahimi Z Vaisi-Raygani A Nagel RL Muniz A Molecular characterization of glucose-6-phosphate dehydrogenase deficiency in the Kurdish population of Western Iran Blood Cells Mol Dis 2006 37 31 37 10.1016/j.bcmd.2006.07.004 16938474

[B28] Karimi M Martinez Di Montemuros F Danielli MG Farjadian S Afrasiabi A Fiorelli G Cappellini MD Molecular characterization of glucose-6-phosphate dehydrogenase deficiency in the Fars province of Iran Haematologica 2003 88 346 347 12651275

[B29] Sukumar S Mukherjee MB Colah RB Mohanty D Molecular basis of G6PD deficiency in India Blood cell Mol Dis 2004 33 141 145 10.1016/j.bcmd.2004.06.003 15315792

[B30] Moiz B Nasir A Moatter T Naqvi ZA Khurshid M Population study of 1311 C/T polymorphism of glucose 6 phosphate dehydrogenase gene in Pakistan - an analysis of 715 X-chromosomes BMC Genet 2009 10 41 19640310 10.1186/1471-2156-10-41 PMC2725355

[B31] Martinez Di Montemuros F Dotti C Tavazzi D Fiorelli G Cappellini MD Molecular heterogeneity of glucose-6-phosphate dehydrogenase (G6PD) variants in Italy Haematologica 1997 82 440 445 9299858

[B32] Menounos P Zervas C Garinis G Doukas C Kolokithopoulos D Tegos C Patrinos GP Molecular heterogeneity of the glucose-6-phosphate dehydrogenase deficiency in the Hellenic population Hum Hered 2000 50 237 241 10.1159/000022922 10782016

[B33] Ainoon O Yu YH Amir Muhriz AL Boo NY Cheong SK Hamidah NH Glucose-6-phosphate dehydrogenase (G6PD) variants in Malaysian Malays Hum Mutat 2003 21 101 12497642 10.1002/humu.9103

[B34] Soemantri AG Saha S Saha N Tay JSH Molecular variants of red cell glucose-6-phosphate dehdrogenase deficiency in central Java, Indonesia Hum Hered 1995 45 346 350 10.1159/000154303 8537082

[B35] Kurdi-Haidar B Mason PJ Berrebi A Ankra-Badu G Al-Ali A Oppenhheim A Luzzatto L Origin and spread of glucose-6-phosphate dehydrogenase variant (G6PD-Mediterranean) in the Middle East Am J Hum Genet 1990 47 1013 1019 1978555 PMC1683892

[B36] Beutler E Kuhl W The NT 1311 polymorphism of G6PD: G6PD Mediterranean mutation may have originated independently in Europe and Asia Am J Hum Genet 1990 47 1008 1012 1978554 PMC1683912

[B37] Tishkoff SA Vasrkonyi R Cahinhinan N Abbes S Argyropoulos G Destro-Bisol G Drousiotou A Dangerfield B Lefranc G Loiselet J Piro A Stoneking M Tagarelli A Tagarelli G Touma EH Williams SM Clark AG Haplotype diversity and Linkage disequilibrium at human G6PD: Recent origin of alleles that confer malarial resistance Science 2001 293 455 462 10.1126/science.1061573 11423617

[B38] Vives Corrons JL Zarza R Aymerich JM Boixadera J Carrera A Colomer D Corbella M Castro M Crespo JM Del Arco A Erkiaga S Font L González I Juncá J Lausin A Manrubia E Martin Núňez G Murga MJ Oliva E Pérez De Mendiguren B Pujades MA Remacha A Rovira A Villegas A Molecular analysis of glucose-6-dehydrogenase deficiency in Spain Sangre (Barc) 1997 42 391 398 9424740

[B39] Kawamoto F Matsuoka H Kanbe T Tantular IS Pusarauati S Kerong HI Damianus W Mere D Dachlan YP Further investigations of glucose-6-phosphate dehydrogenase variations in Flores Island, eastern Indonesia J Hum Genet 2006 51 952 957 10.1007/s10038-006-0044-y 16927025

[B40] Nafa K Reghis A Osmani N Baghli L Benabadji M Kaplan JC Vulliamy TJ Luzzatto L G6PD Aures: a new mutation (48 Ile- > Thr) causing mild G6PD deficiency is associated with favism Hum Mol Genet 1993 2 81 82 10.1093/hmg/2.1.81 8490627

[B41] Al Jaouni SK Molecular clinical correlation of glucose 6-phosphate deficiency in western Saudi Arabia Haematologica 2006 91 S1 24 2516434367

[B42] Al-Ali AK Common G6PD variant from Saudi population and its prevalence Ann Saudi Med 1996 16 654 656 17429252 10.5144/0256-4947.1996.654

[B43] Al Arrayed S Campaign to control genetic blood diseases in Bahrain Community Genet 2005 8 52 55 10.1159/000083340 15767757

[B44] Usanga EA Ameen R Glucose-6-phosphate dehydrogenase deficiency in Kuwait, Syria, Egypt, Iran, Jordan and Lebanon Hum Hered 2000 50 158 161 10.1159/000022906 10686492

[B45] El Fakhri M Glucose-6-phosphate dehydrogenase deficiency in Libya: an appraisal of gene frequency in Arabic regions of Africa and West Asia Garyounis Med J 1985 8 143 146

[B46] Al-Riyami A Ebrahim GJ Genetic Blood Disorders Survey in the Sultanate of Oman J Trop Pediatr 2003 49 Suppl 1 i1 i20 12934793

[B47] Alabdulaali MK Alayed KM Alshaikh AF Almashhadani SA Prevalence of glucose-6-phosphate dehydrogenase deficinecy and sickle cell trait among blood donors in Riyadh Asian J Transf Sci 2010 4 31 33 10.4103/0973-6247.59389 PMC2847342 20376264

[B48] Warsy AS El-Hazmi MAF G6PD deficiency, distribution and variants in Saudi Arabia: an overview Ann Saudi Med 2001 21 174 177 17264545 10.5144/0256-4947.2001.174

[B49] Omer A Ali M Omer AH Mustafa MD Satir AA Samuel AP Incidence of G-6-PD deficiency and abnormal haemoglobins in the indigenous and immigrant tribes of the Sudan Trop Geogr Med 1972 24 401 405 4648653

[B50] Altay C Gümrük F Red Cell glucose-6-phosphate dehydrogenase deficiency in Turkey Turk J Hematol 2008 25 1 7 27264143

[B51] Clark TG Fry AE Auburn S Campino S Diakite M Green A Richardson A Teo YY Small K Wilson J Jallow M Sisay-Joof F Pinder M Sabeti P Kwiatkowski DP Rockett KA Allelic heterogeneity of G6PD deficiency in West Africa and severe malaria susceptibility Eur J Hum Genet 2009 17 1080 1085 10.1038/ejhg.2009.8 19223928 PMC2986558

[B52] Bouanga JC Mouélé R Préhu C Wajcman H Feingold J Galactéros F Glucose-6-phosphate dehydrogenase deficiency and homozygous sickle cell disease in Congo Hum Hered 1998 48 192 197 9694250 10.1159/000022801

[B53] Carter N Pamba A Duparc S Waitumbi JN Frequency of glucose-6-phosphate dehydrogenase deficiency in malaria patients from six African countries enrolled in two randomized anti-malarial clinical trials Malaria J 2011 10 241 10.1186/1475-2875-10-241 PMC3188486 21849081

[B54] Khneisser I Adib SM Loiselet J Megarbane A Prevalence of G6PD deficiency and knowledge of diagnosis in a sample of previously unscreened Lebanese males: clinical implications J Med Screen 2006 13 26 28 10.1258/096914106776179827 16569302

[B55] Luzzatto L Notaro R Malaria. Protection against bad air Science 2001 293 442 443 10.1126/science.1063292 11463901

[B56] Morsy H Mokhtar M Nazmy N El-Gezeery A Abdulla E Neonatal screening and molecular genetic characterization of glucose-6-phosphate dehydrogenase deficiency in Alexandria, Egypt HUGO J 2011 5 229

